# Unearthing the Fossorial Tadpoles of the Indian Dancing Frog Family Micrixalidae

**DOI:** 10.1371/journal.pone.0151781

**Published:** 2016-03-30

**Authors:** Gayani Senevirathne, Sonali Garg, Ryan Kerney, Madhava Meegaskumbura, S. D. Biju

**Affiliations:** 1 Department of Molecular Biology & Biotechnology, Faculty of Science, University of Peradeniya, Peradeniya, Sri Lanka; 2 Systematics Lab, Department of Environmental Studies, University of Delhi, Delhi, India; 3 Department of Biology, Gettysburg College, Gettysburg, Pennsylvania, United States of America; Smithsonian's National Zoological Park, UNITED STATES

## Abstract

Tadpoles of the monotypic Indian dancing frog family Micrixalidae have remained obscure for over 125 years. Here we report the discovery of the elusive tadpoles of *Micrixalus herrei* from the sand beds of a forested stream in southern Western Ghats, and confirm their identity through DNA barcoding. These actively burrowing tadpoles lead an entirely fossorial life from eggs to late metamorphic stages. We describe their internal and external morphological characters while highlighting the following features: eel-like appearance, extensively muscularized body and tail, reduced tail fins, skin-covered eyes, delayed development of eye pigmentation in early pre-metamorphic stages (Gosner stages 25–29), prominent tubular sinistral spiracle, large transverse processes on vertebrae II and III, ankylosed ribs on transverse processes of vertebra II, notochord terminating before the atlantal cotyle-occipital condyle junction, absence of keratodonts, serrated well-formed jaw sheaths, and extensive calcified endolymphatic sacs reaching sacrum posteriorly. The tadpole gut contains mostly fine sediments and sand. We discuss the eel-like morphology and feeding habits of *M*. *herrei* in the context of convergence with other well-known fossorial tadpoles. This discovery builds the knowledge base for further comparative analyses and conservation of *Micrixalus*, an ancient and endemic lineage of Indian frogs.

## Introduction

Amphibians portray a diversity of reproductive strategies for a life on land and water, which often includes presence of an aquatic tadpole and a more terrestrial adult form following metamorphosis [1]. This biphasic life history mode of extant amphibians (caecilians, urodeles and anurans) is considered plesiomorphic and widespread [2–4]. Among the 54 families of anurans [5], only tadpoles of family Micrixalidae (Dubois, Ohler & Biju 2001) remain unknown. Micrixalidae is endemic to the Western Ghats region of India and currently contains only one genus, *Micrixalus* Boulenger, 1888, an ancient frog lineage that diversified during the Upper-Cretaceous or Paleocene period [6]. The type species of the genus (and family), *Micrixalus fuscus* (Boulenger 1882), was described over 125 years ago. Adult *Micrixalus* mostly associate with fast-flowing perennial streams, implying that they also breed in or near these streams [7,8]. Given the local abundance of some species, their consistent habitat associations, and their undisputed recognition as members of a unique anuran lineage, it is surprising that the tadpoles of these frogs remained unknown until now.

Previous studies of *Micrixalus* systematics and reproductive behavior provided vital information leading to the discovery of its tadpoles. The recent increase in recognized diversity from 11 [9] to 24 species [8], affirmed that micrixalid taxonomy has been under-studied. These frogs, however, are well known for their amplexus and unique sexual display, which involves foot-flagging or “dancing” of male frogs on boulders in forested streams [7–13].

*Micrixalus herrei* Myers, 1942 was recently resurrected from the synonymy of *M*. *fuscus* [8]. Amplexed females of this species lay eggs in sandy depressions made in shallow streams during the pre- and post-monsoon periods. This depression is then completely covered with sand by the female [7,8]. Though the fate of eggs from this point onwards is unknown, these observations suggest that at least the initial developmental stages of this species are fossorial, which may explain why the tadpole and its habits were unknown. The stream association of *M*. *herrei*, and apparent absence of its free-swimming tadpoles in the water column, led us to explore the stream substrate to discover the fossorial larval stages of this species.

A cursory description of two “poorly preserved” *Micrixalus* tadpoles was provided by Smith [14] and mentioned in some subsequent studies [15–17]. However, Smith [14] did not provide information about the habits and habitats of those tadpoles, did not accession his specimens, and likely incorrectly identified his material (see discussion).

Tadpoles exhibit morphological convergence in habitat and feeding behaviors [1]. Fossoriality, broadly defined as burrowing in mud or sand, or living among gravel beds, is one of the 20 ecomorphological guilds of tadpoles [1,18,19]. Globally, only a few families with fossorial tadpoles are known or inferred. These include members of the families Centrolenidae, Megophryidae, Microhylidae, Ranidae, and Arthroleptidae (see S1 Table for references).

Eel-like, “anguilliform,” bodies are characteristic of fossorial larvae (e.g., 20–23). Some of these forms like centrolenids, ranids, and arthroleptids seek refuge under leaf litter and mulch along streams, but most megophryids actively burrow into sand and gravel beds. Furthermore, depending on their habitat, these larvae often possess specialized morphological adaptations for feeding [21–23]. Feeding is a constraint that all these larvae face while hidden in these habitats. Some specialize as “sand-eaters.” These feed on organic matter found among sand grains [24–26]. Since it is impossible to separate only organic particles from sand using the mouth, they inadvertently ingest sand grains together with the organic matter.

*Otophryne pyburni*, a psammonic tadpole (i.e., tadpoles that feed while sand burrowing [1]), uses a passive filter feeding mechanism despite being fossorial [21,24]. Additionally, *Scaphiophryne gottlebei* and *Boophis picturatus* are also known to ingest sand and the associated organic material, however, neither is fully fossorial. *Scaphiophryne gottlebei* is closely associated with streambeds, and burrows half of its body within sand during the daytime and swims within the water column at night [25], whereas *Boophis picturatus* is mostly found on sand [24,26], and examinations of their gut contents have revealed that they are also “sand-eaters” [26].

Following extensive explorations of the lotic microhabitats, we show that *Micrixalus herrei* tadpoles are completely fossorial and possess specialized morphological features for their unusual lifestyles. We describe the life history, external morphology, and osteology of these tadpoles. Furthermore, we discuss the evolutionary convergence associated with this life history mode by comparing with other known fossorial tadpoles.

## Materials and Methods

### Ethics statement

This study was conducted with permissions and guidelines from the responsible authorities in the State Forest Department of Kerala. The protocol of our collection and research complied with the ethical conditions outlined in the Wildlife (Protection) Act 1972, Government of India. Specific methods of collection, euthanasia, tissue sampling and fixation followed the guidelines for use of live amphibians and reptiles in field research by the American Society of Ichthyologists and Herpetologists (ASIH) (http://www.asih.org/pubs/herpcoll.html; dated 13 March 2006), and were approved by the internal ethical committee of Department of Environmental Studies, University of Delhi.

### Field surveys

Fieldwork by SD Biju (SDB) in the Western Ghats, and recent findings of Biju *et al*. [8] helped establish the potential areas of streams for focused exploration of tadpoles of *Micrixalus*. The tadpole of *M*. *herrei* was first observed by SDB in 2004, at Chathankod–Bonnacaud area (08.6737° N 77.1575° E, 488 m asl), located at the base of Agasthyamalai hills in the Western Ghats state of Kerala. A dedicated survey was later conducted in the same locality during the months of January and February 2012, and the search specifically focused on streambeds. We surveyed a perennial stream of second to third order, located inside a primary moist deciduous forest, where *M*. *herrei* was abundant. The specific survey site at the stream was covered with a closed forest canopy. Tadpole behavior and development were studied in the field as well as in captivity. Efforts were concentrated towards searching the gravel and sand beds along the shallow water on the sides of streams, which were previously observed as sites of egg-laying [8].

### Field and captive studies

Daytime (10:00–17:00 hours) searches were made along streambeds to locate tadpoles of *Micrixalus herrei*. Microhabitats within the stream (bottom, pools and edges) and stagnating pools beside the stream were initially searched for tadpoles using pond-nets (20 person-hours). Wider pools were seined by two people using a 1.5 x 1 m net (10 person-hours). Subsequent efforts were directed to stream sand beds; 120 person-hours were spent digging multiple pits to a maximum depth of 2 feet. The behavior of the tadpoles was observed in the field as soon as they were unearthed.

Initial development of an egg clutch was studied under captive conditions. A freshly laid egg clutch (*N* = 20) was collected (23 October 2012 at 16:00 hours, from depths between 15–20 mm) from the survey site soon after a female covered it with sand and left the site. Characteristics of the egg-laying habitat and the nest were noted. The clutch was then carefully removed by exposing the oviposition site and placed in a glass aquarium along with sand, gravel, and water from the capture site (water temperature 22°C). Sand and water level above the egg clutch were simulated in captivity. Observations were made on alternate days by careful shifting of sand and gravel.

### Tadpole identification by DNA barcoding

Fossorial tadpoles (*N* = 26) believed to be *Micrixalus herrei*, were euthanized in tricaine methanesulfonate (MS-222). A specimen was preserved in absolute ethanol, and stored at –20°C in the Systematics Lab, University of Delhi (SDBDU) for species verification. DNA was extracted from ethanol-preserved tail muscle tissue using a standard protocol [27]. A 540 bp fragment of the mitochondrial 16S ribosomal RNA was PCR-amplified following primers and protocol of Simon *et al*. [28]. Sanger sequencing was performed on both strands using an ABI 3730 automated DNA sequencer (Applied Biosystems). Sequences were assembled and edited in ChromasPro v1.34 (Technelysium Pty Ltd.). Preliminary identification of the taxon was done by conducting BLAST searches against a near-complete database of sequences of *Micrixalus* species. Subsequently, the generated sequence was compared against a dataset consisting of *Micrixalus herrei* (KJ711301, closest hit of the BLAST search) and other micrixalid species that were reported to occur in the same area [8], *M*. *fuscus* (KJ711283), *M*. *mallani* (KJ711324) and *M*. *sali* (KJ711354). ClustalW, as implemented in MEGA 5.0 [29], was used to align the sequences. MEGA was also used to calculate uncorrected pairwise divergences. Newly generated sequence was deposited in GenBank under the accession number KU833220.

### External morphology

Freshly collected tadpoles of different developmental stages were euthanized using MS-222 and fixed in 10% neutral-buffered formalin. These were staged according to Gosner [30] and described following the external morphological terminology of McDiarmid & Altig [18], Altig [31], and Grosjean [32]. External morphological measurements were made using a digital caliper, or a binocular microscope with a micrometre ocular, to the nearest 0.01 mm. Abbreviations of the measurements include: maximum height of body (BH), maximum length of body (BL), maximum width of body (BW), maximum diameter of eye (ED), extent of eye pigmentation (EP) = diameter of the pigmented-portion of the eye, internarial distance (NN), naro-pupillary distance (NP), interpupillary distance (PP), rostro-narial distance (RN), distance from tip of snout to opening of spiracle (SS), distance from tip of snout to insertion of upper tail fin (SU), tip of snout to opening of the vent tube (SVL), total length (TL), distance from vent to tip of tail (VT), tail muscle height (TMH) and tail muscle width (TMW). Tadpoles in late metamorphic stages were identified as that of *M*. *herrei* based on Biju *et al*. [8]. Tadpoles are deposited in the Zoological Survey of India–Western Ghats Regional Station (ZSI/WGRS), Kozhikode under the accession number ZSI/WGRC/V/A/907 and Systematics Lab, University of Delhi (SDBDU).

### Internal morphology

Buffered formalin-preserved tadpoles of developmental stages 36, 42 and 46 were cleared and differentially stained for bone and cartilage using the procedures of Hanken & Wassersug [33] and Taylor & Van Dyke [34]. Chondrocranial terminology follows that of Cannatella [35]. Osteological terminology follows Trueb [36] and Duellman & Trueb [37]. Cleared and double-stained specimens are deposited in the Systematics Lab, University of Delhi (SDBDU) under the accession number SDBDU 2012.2448. A freshly preserved developmental series of larvae (*N* = 8; stages 25, 28, 30, 36, 38, 40, 42 and 45) was dissected to observe gut contents.

## Results

### Genetic identification of the tadpoles

Based on the uncorrected pairwise distance results, DNA sequence of the discovered tadpole was identified as that of *Micrixalus herrei* and had a pairwise divergence of 0.5% with the available adult sequence. Compared against other *Micrixalus* species, the tadpole sequence showed considerable divergence: 5.6% with *M*. *fuscus*, 4.8% with *M*. *mallani*, and 8.0% with *M*. *sali*.

### Tadpole ecology and behavior

**Fig 1:**
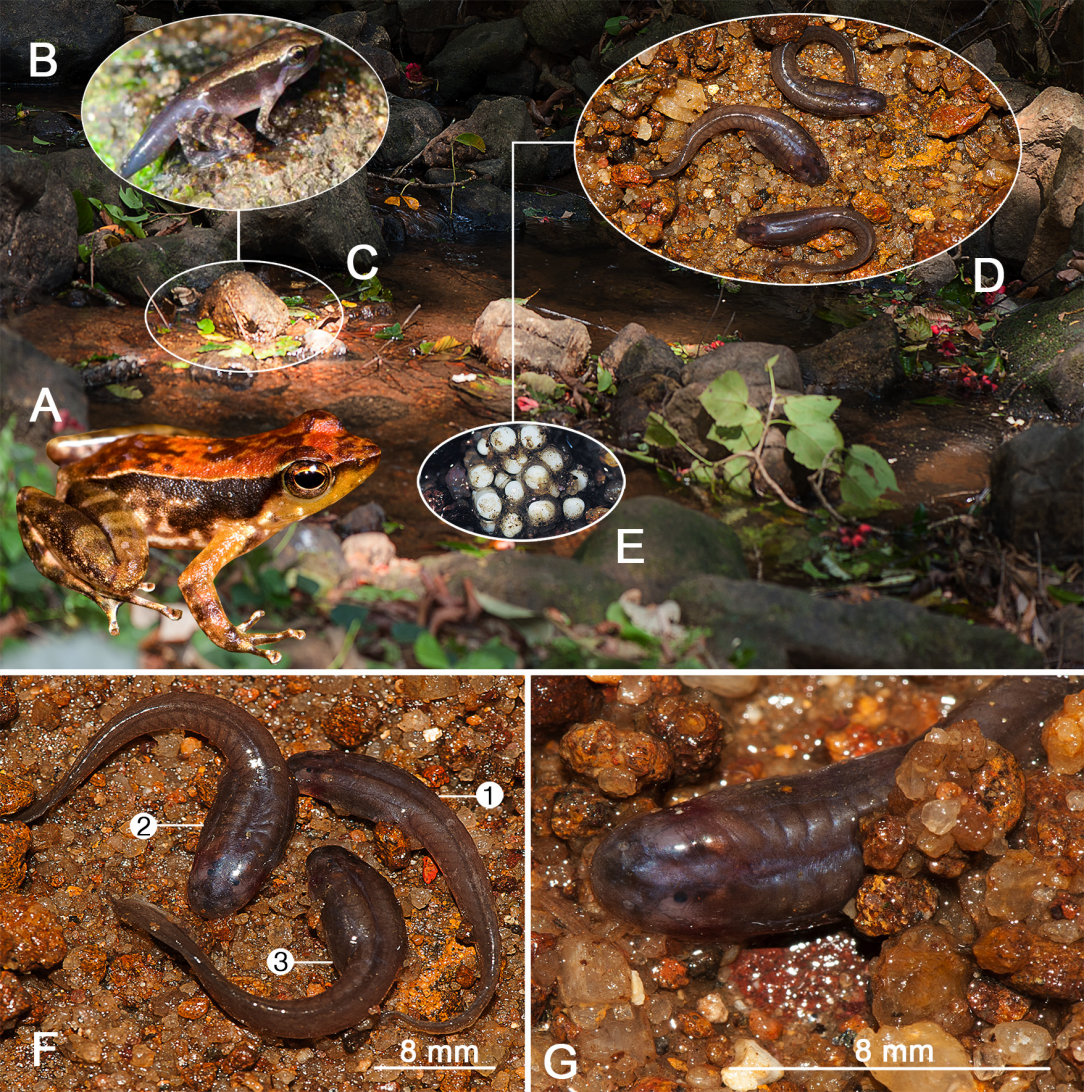
Habitat preference and characteristic features of various life history stages of *Micrixalus herrei*. (A) Adult male (SDBDU 2012.2452, SVL 17.40 mm), found on the surface of emergent wet rocks along the stream; (B) metamorphosed larva, near shallow water, closer to the stream bank (stage 44, SVL 17.20 mm); (C) stream margin habitat of metamorphs (stages 43–44); (D) anguilliform bodied tadpoles on the gravel bed (stages 30–35), after being exposed from depths of about 30 cm; (E) egg clutch (*N* = 20) buried under sand in shallow water; (F) fossorial tadpoles after being exposed, at stages 26 (1), 28 (2), and 29 (3), having eel-like, dorsoventrally flattened bodies, dorsal eyes, and well-developed muscular tails with reduced fins; (G) lateral profile of a tadpole, observed amongst exposed gravel (stage 27).

Careful searching through streambed sand, gravel, and stones resulted in the first validated discovery of eel-like tadpoles of *Micrixalus herrei*. Eight tadpoles were observed (stages 26–33) within an hour of digging. Each wriggled back into the gravel bed upon exposure, defying capture. Further digging and rapid action led us to collect 13 fossorial tadpoles of various sizes and developmental stages (Fig 1A–1E and Table 1). These were collected from sand and gravel beds along the edges of the forest stream covering an area of approximately 100 m^2^. Tadpoles were found at depths between 10 and 40 cm. Larger aggregations of tadpoles (between 4 and 8 individuals, Fig 1D and 1F) often occupied larger interstitial spaces among submerged pebble formations. Solitary tadpoles were commonly found among finer-sized sand and smaller pebbles (Fig 1G). Behavioral observations in the wild showed that exposed tadpoles always tried to escape immediately and quickly by burrowing into sand using vigorous eel-like movements. They were even observed burrowing into wet sand in the absence of free water, confirming their active and potentially obligate burrowing nature. They were never seen free-swimming in open water or resting at the bottom of the stream. Metamorphic stages with externally visible well-developed limbs (stages 42–43) were observed on the banks, away from stream edges. The preferred microhabitat of metamorphs appears to be away from water (Fig 1B and 1C). Froglets (stages 44–46; SVL 10–12 mm, *N* = 5) were always observed on leaf litter or small pebbles near the stream banks.

**Table 1:** Mean values of external morphological measurements of *Micrixalus herrei* tadpoles (*N* = 2 from each stage) at different stages.

	Lateral measurements								Dorsal measurements					
Stage	BH	ED	SU	SVL	SS	VT	TMH	TL	BW	TMW	PP	NN	RN	NP
25	3.14	0.48	5.1	5.89	2.67	10.13	2.38	16.02	2.21	1.47	1.34	0.82	1.96	1.51
26	3.88	0.72	6.22	6.45	2.97	10.69	2.74	17.14	2.62	1.74	1.49	0.95	1.9	1.77
27	4.53	0.76	8.96	9.66	3.07	11.72	3.31	21.38	3.80	2.53	1.62	1.87	1.82	1.83
28	3.48	1.24	9.42	9.75	3.18	11.66	3.67	21.41	3.98	2.65	1.78	0.98	1.98	1.91
29	3.66	1.88	9.18	9.26	4.01	14.95	4.35	24.21	4.37	2.91	1.39	1.5	2.02	1.92
30	4.12	2.28	9.12	10.77	5.16	15.52	4.41	26.29	4.47	2.98	1.63	1.96	2.4	1.48
31	3.56	2.98	9.46	10.67	5.22	18.23	4.65	28.9	5.03	3.35	1.83	1.93	2.00	1.61
32	3.31	3.01	10.14	10.50	5.21	19.22	4.71	29.72	5.19	3.46	1.20	1.56	1.92	1.14
33	3.52	3.04	10.55	10.91	5.25	19.76	4.75	30.67	5.23	3.48	1.90	1.76	2.2	1.35
35	3.66	3.24	10.95	11.34	5.29	19.69	4.57	31.03	5.52	3.68	1.72	1.74	1.34	1.27
36	4.2	3.64	11.43	11.76	5.59	19.64	4.43	31.4	5.64	3.76	1.86	1.98	2.3	1.26
37	3.01	3.12	12.3	12.59	5.72	20.13	4.64	32.72	5.70	3.80	1.62	1.53	2.02	1.02
38	4.15	4.56	12.2	12.62	5.44	21.17	4.83	33.79	5.87	3.91	2.34	1.78	2.06	1.85

Measurements are given in mm.

The cavity in the streambed in which the female deposited eggs (Fig 1E) was between 15–20 mm deep and the height of water column above the cavity was between 20–25 mm. Freshly laid eggs were creamy-white in color and measured 0.8–1.2 mm, *N* = 20 (Fig 1E). Eggs hatched after seven days in captivity under field conditions (housed in a glass aquarium; using sand and water from the stream; water was replaced regularly; water temperature was 22°C). Tadpoles were creamy white (stage 20, TL = 13.20 mm) and remained within sand and gravel with minimal movement. However, no further tadpole development was observed over the next 15 days in captivity, after which they died from unknown causes. This premature death may be due to the temperature changes or lack of nutrients in captive conditions.

**Fig 2:**
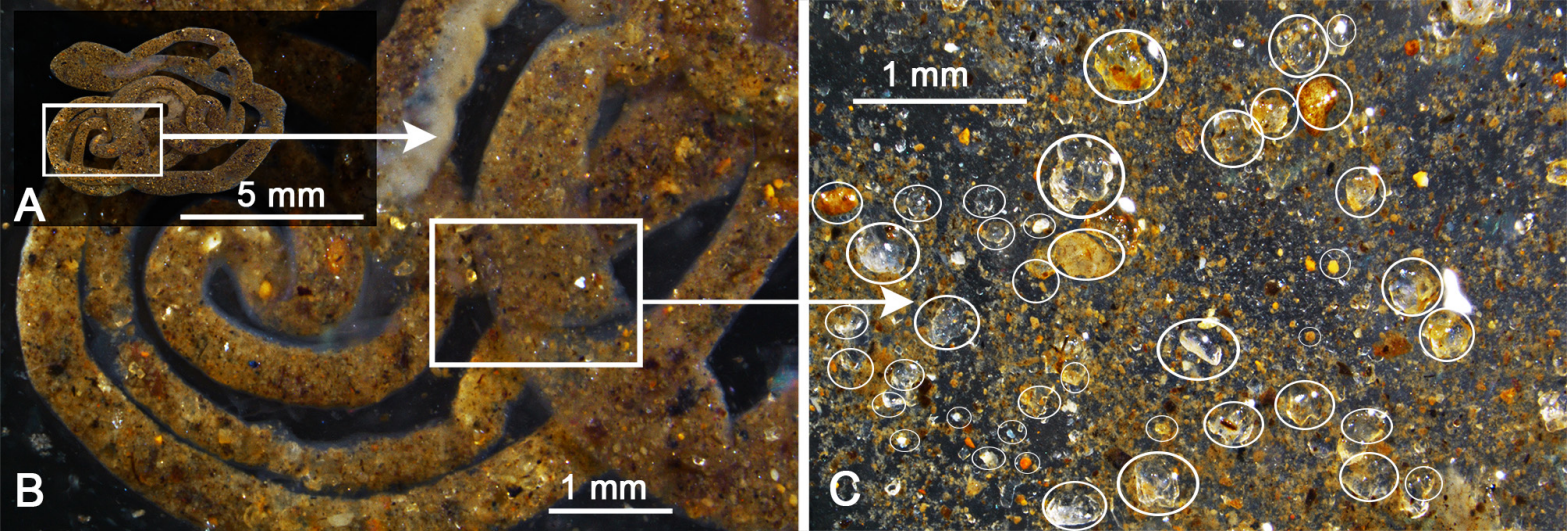
Gut contents of *Micrixalus herrei* tadpole (stage 37). (A) Coiled gut with a narrower anterior region and a wider rear end, completely filled with sand, 9.7 cm long; (B) middle part of the gut filled with sand and organic material; (C) magnified view of various sized sand grains (particles ranging between 0.1–0.7 mm, with a few large sand particles indicated within circles) and sediment particles observed inside the coiled intestines.

Our overall understanding of the behavior and fossorial nature of *Micrixalus* tadpoles is largely based on observations in the wild since complete tadpole development was not achieved under captive conditions. Dissection of guts (Fig 2A–2C) revealed the presence of sand grains (maximum width ranging between 0.1–0.7 mm) through the whole length of the coiled-intestines of stage 25–40 tadpoles collected from the field, irrespective of their size.

### Diagnostic external morphology of the tadpole

**Fig 3:**
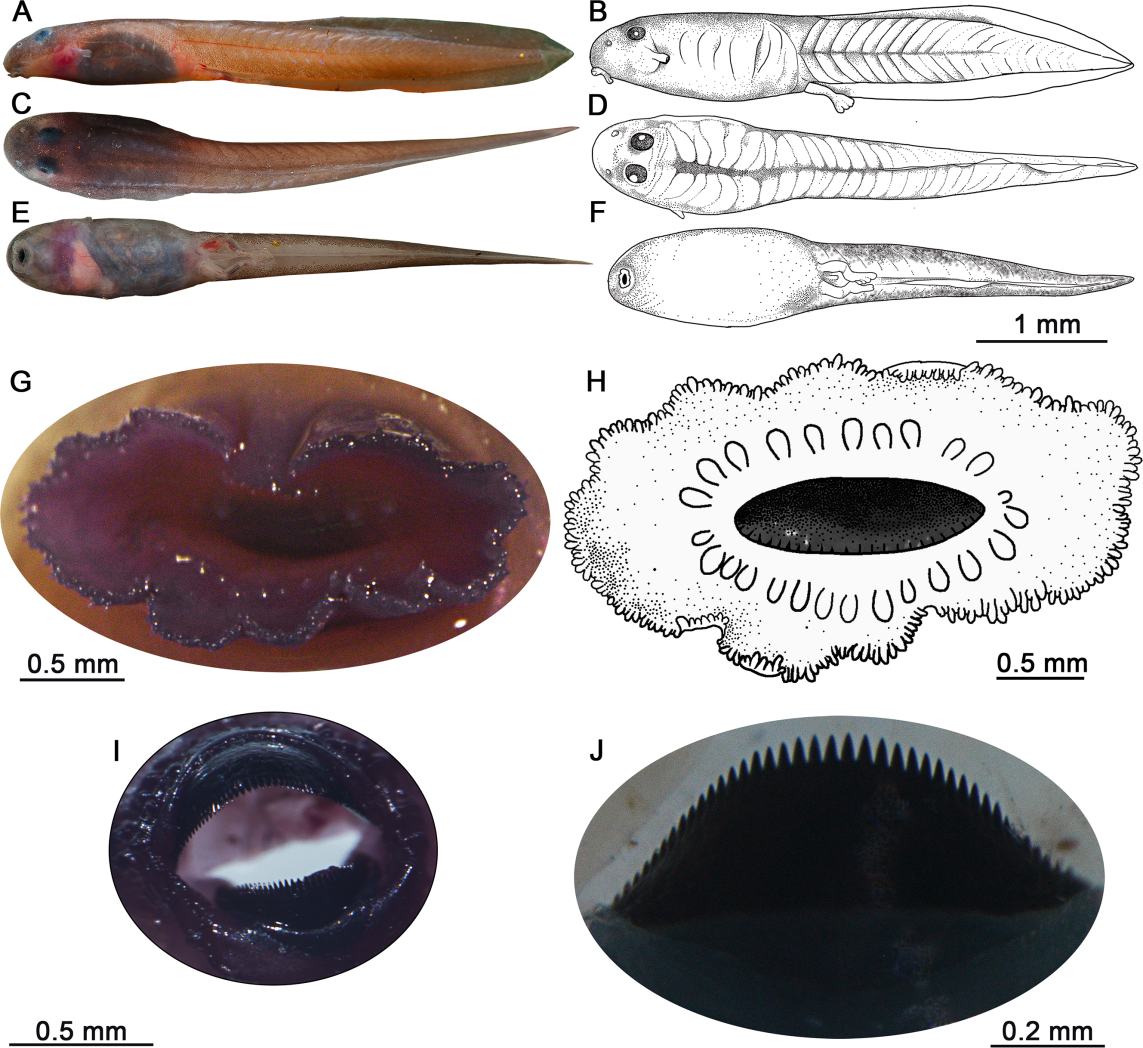
External and oral morphology of *Micrixalus herrei* tadpoles (stage 36). (A, B) Lateral profile, exhibiting tubular spiracle and skin covered well-pigmented eyes; (C, D) dorsal profile, showing the anguilliform body and robust epaxial musculature; (E, F) ventral profile, exhibiting funnel-shaped oral disc, dextral vent tube and developed hindlimbs; (G, H) funnel-shaped oral disc, with its margins demarcated by a single row of blunt marginal papillae and elongated submarginal papillae; (I) large, robust upper and lower jaw sheaths covering the mouth opening; (J) lower jaw sheath with pointed serrations bordering the anterior margins.

**Fig 4:**
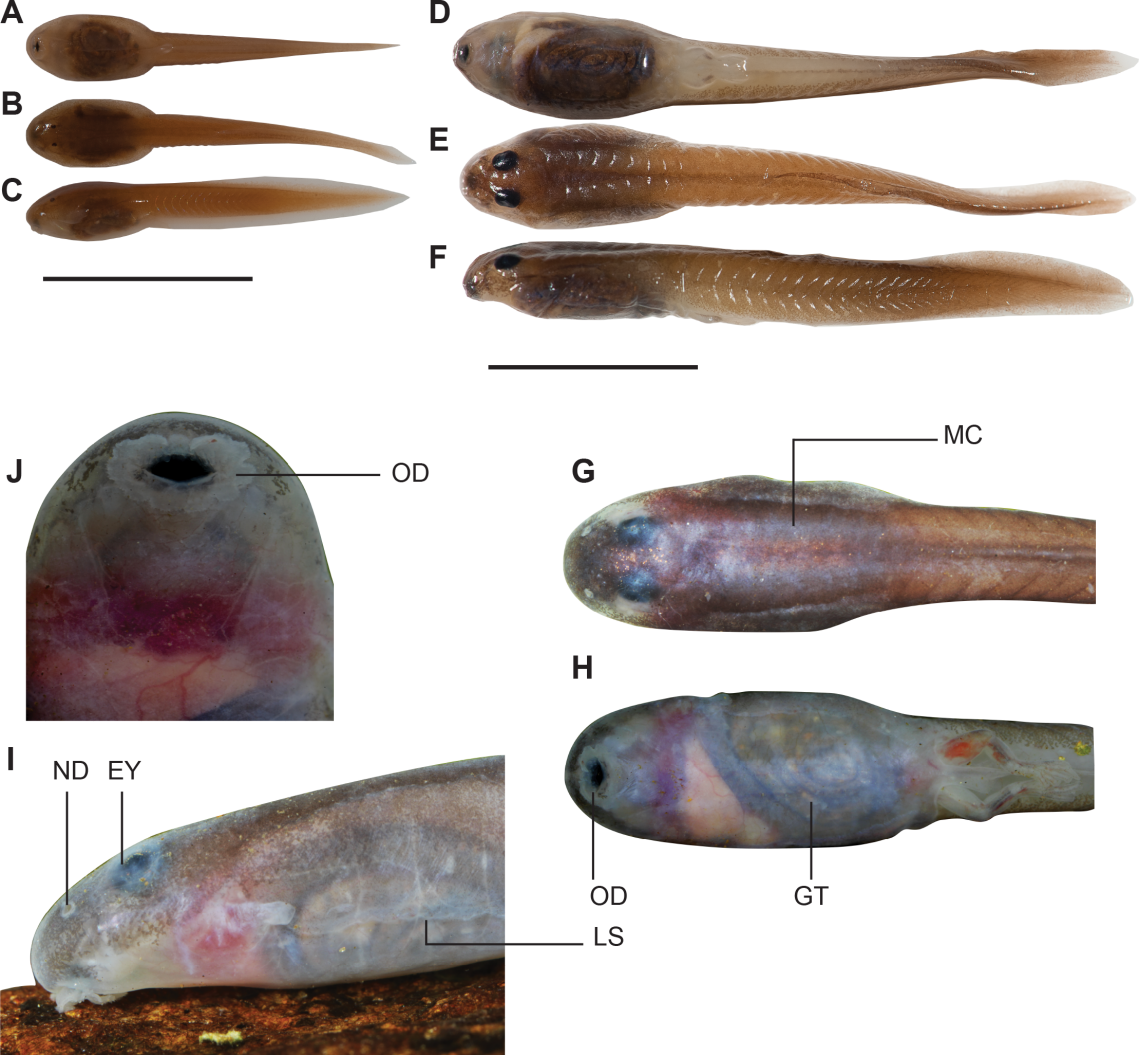
Ontogenetic variation of *Micrixalus herrei*. (A) Ventral view, (B) dorsal view, (C) lateral view of a stage-25 tadpole with poor eye development; (D) ventral view, (E) dorsal view, (F) lateral view of a stage-37 tadpole with well-developed eyes; (G) dorsal view, (H) ventral view of the body region at stage 36; (I) lateral profile at stage 36; (J) elliptical, funnel-shaped oral disc demarcated by continuous marginal papillae at stage 36. Abbreviations: EY, eyes overlaid with thickened skin; GT, coiled gut filled with sand sediments; LS, lateral expansion of skin; MC, well-muscularized tail; ND, narial depression; OD, oral disc. Scale bars: 10 mm.

Descriptions are based on stage 36 tadpoles (*N* = 3) of *Micrixalus herrei* (Fig 3A–3F) except when explicitly stated otherwise: body relatively long (BL 32% of TL) having a narrower anterior region and a slightly wider posterior region due to the coiled intestine, elliptic in dorsal profile (Fig 3C and 3D), depressed shape in lateral profile (Fig 3A and 3B); marginal separation of body and tail is not clear in some specimens in dorsal profile; bulbous eyes (ED/BW = 0.16) positioned dorsally, covered with thickened skin (Figs 3A, 3C, 4G and 4I); elygium absent; nasolacrimal duct absent; ventral side of the body often appears red-tinged around the center with apparent capillary network beneath the skin (Fig 3E); snout appears rounded when viewed both dorsally and laterally (Fig 3A–3D); non-perforated narial depressions are elliptical, located more laterally than anterolaterally, closer to eyes than the snout (Fig 3A and 3B); pigmentation is visible around the closed narial depressions; sinistral spiracle tube-like, clearly protruding from the body, located closer to snout than posterior margin of body (Fig 3A and 3B); spiracular opening located at an average height of 6.01 mm (53% of SVL and 39% of BH) from the ventral margin of body; coiled gut moderately long (9.7 cm) with 5–6 loops, filled with sand and sediments (Figs 2A–2C and 3E).

The tail is also unique for fossorial tadpoles: well-developed tail musculature arises at the first third of the body region and extends almost to the tail tip (Fig 3A–3D), separated by V-shaped septa (TMH 84% of BH and TMW 59% of BW); dorsal and ventral tail fins are of equal length, reduced (Fig 3A–3D); tail fin is wider anteriorly and narrows as it reaches the tail tip, ending bluntly; posterior tapering of the tail fins begins at the first third of the tail region; point of maximum height of tail is located just before the midsection; tubular vent is posteriorly positioned, dextral and well developed (Fig 3E and 3F).

### External body coloration (in life)

Tadpoles are colored with dense light brown pigments on the dorsum. Increased density of pigments occurs anterior to the narial depressions. Silver iridiophores are observed between the eyes and along the dorsal midline region of the body. Ventral abdominal region is translucent, with a red tinge. The sand-filled coiled gut is clearly visible beneath the translucent skin. The ventral oral disc is transparent, while hindlimbs are translucent with visible capillary networks. Dorsal and ventral tail membranes are light grey and the tail musculature is pigmented light brown throughout. Membrane and musculature pigment density decrease posteriorly.

During early development (stage 25) in *Micrixalus herrei*, bulbous eyes are prominent (Figs 1A, 1D and 4). However, the eyes have little pigmentation (poorly developed), with EP being only 38–48% of the total eye diameter (ED). Gradual development of eye pigmentation is seen as the development progresses. Pigmentation covers 70% and 100% of the eyes by stage 30 and 34, respectively.

### Oral morphology of the tadpole

Description of the oral morphology is based on stage 36 (*N* = 3) tadpoles. The oral disc is elliptical in shape (Fig 3G and 3H). Width of the disc is 57% of body width (BW). The margin of the oral disc is demarcated by a row of uniserial and blunt marginal papillae that create a wavy edge. These marginal papillae are continuous except for a small medial gap on the upper labium (Fig 3G and 3H). Emarginations are not present. Submarginal papillae are on average twice as long as the marginal ones but are fewer in number. These are present on both anterior and posterior labia between the lateral ends of upper and lower jaws (Fig 3G and 3H). Upper and lower jaws (Fig 3I and 3J) assume inverted U and V-shapes respectively (Fig 3I). These keratinized and robust jaw sheaths have comb-like serrations on their cutting edges (Fig 3I and 3J), however, keratodonts (labial tooth rows) and tooth ridges are absent.

### Chondrocranium of the tadpole

**Fig 5:**
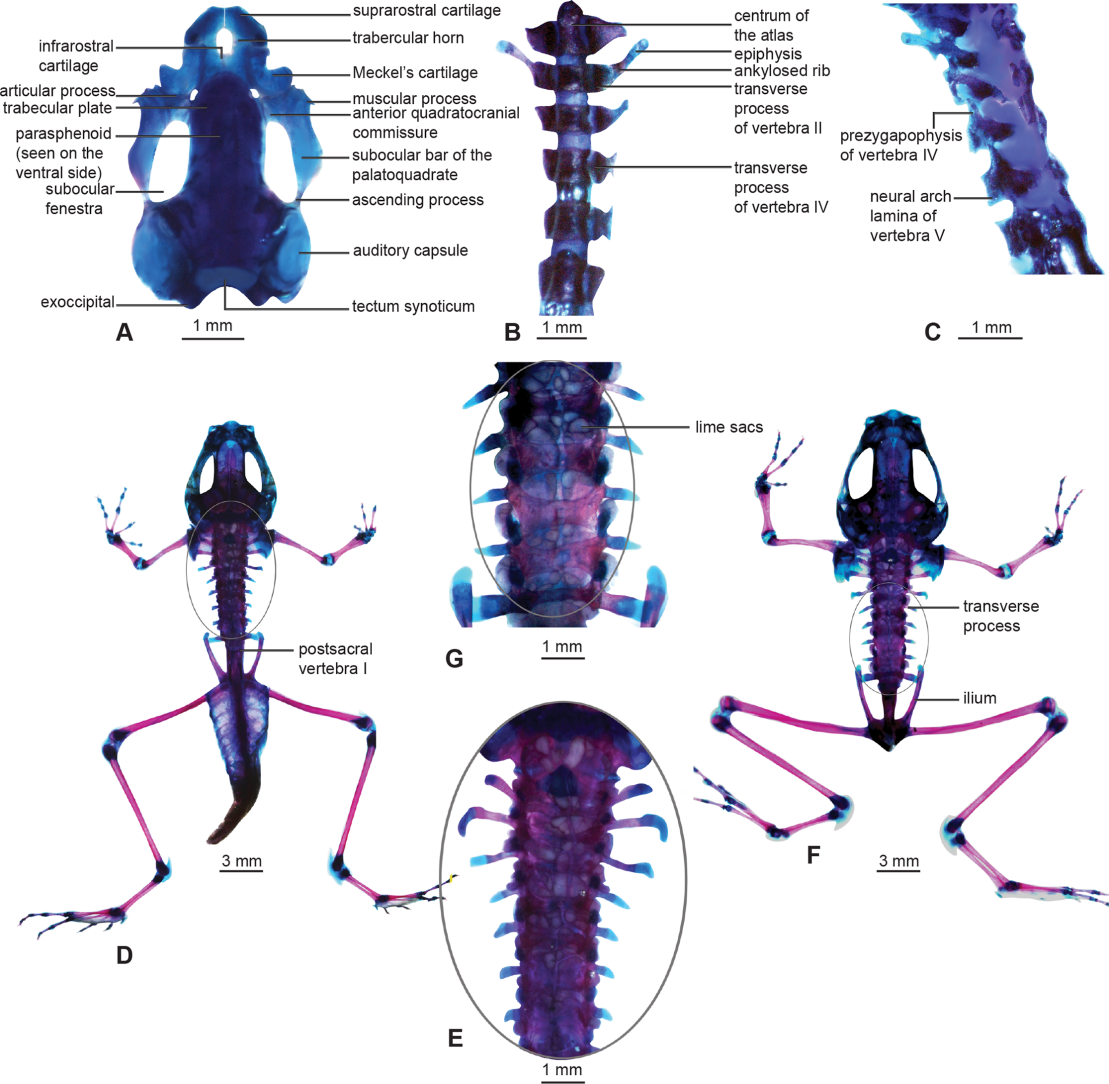
Double-stained specimens of *Micrixalus herrei* life history stages. (A) Chondrocranium, dorsal view at stage 36; (B) vertebral column, ventral view at stage 36; (C) vertebral column, lateral view at stage 36; (D, E) stage 42 and (F, G) stage 46 metamorphs with lime sacs extending along the extradural space of the vertebral column.

Description of the larval chondrocranium is based on stained-specimens of stage 36 (*N* = 2). The chondrocranium is narrow and oblong-shaped (Fig 5A). The maximum width is 67.34% of its total length and its widest part is at the posterior end of subocular bar of the palatoquadrate (Fig 5A). The jaw suspension creates an angle of 30° with the floor of the neurocranium resulting in a relatively flattened skull.

In most tadpoles, the palatoquadrate is connected to the neurocranium via three processes—anterior quadratocranial commissure, ascending process, and otic process [35]. In *M*. *herrei*, the palatoquadrate attaches to the neurocranium through only two processes: a stout anterior quadratocranial commissure attaches to the neurocranium anteriorly and a thin ascending process joins it posteriorly via an attachment to the orbital cartilage.

The palatoquadrate is wider at its anterior end (Fig 5A). The subocular bar is posterolaterally rounded prior to the ascending process. A poorly developed muscular process extends dorsally from the anterior end of the palatoquadrate and possesses a flattened posterior end and a pointed anterior tip. The anterior margin of the articular process attaches to the posterior margin of Meckel’s cartilage.

The trabecular plate forms the anterior wall of the neurocranium and gives rise to divergent, flattened, narrow and short trabecular horns. These horns extend anteriorly parallel to one another (Fig 5A). The suprarostral cartilages are robust, and they attach to the ventral half of the trabecular horns through multiple articulations. Paired suprarostral cartilages are not fused with one another medially and appear as an inverted U-shape when viewed dorsally (Fig 5A). The large frontoparietal fenestra is bordered by the trabecular plate anteriorly, orbital cartilages laterally, and tectum synoticum posteriorly.

The lower jaw is made up of robust Meckel’s cartilages laterally and infrarostral cartilages medially (Fig 5A). Meckel’s cartilages are L-shaped and oblique to the longitudinal axis of the chondrocranium. They articulate with the infrarostrals dorsomedially. Infrarostrals are U-shaped when viewed anteriorly and are connected to each other via a symphysis medially.

### Morphology of the vertebral column at stages 36, 42 and 46

At stage 36, the vertebral column consists of eight presacral vertebrae, one sacral vertebra, and a single postsacral vertebra. The atlas has two prominent atlantal cotyles (Fig 5B), which articulate with the occipital condyles. The notochord terminates at this atlantal cotyle-occipital condyle joint and extends posteriorly towards the tip of the tail. Vertebrae II–VI bear paired prezygapophyses and postzygapophyses (Fig 5C) on the anterior and posterior ends, respectively. The atlas only bears a pair of postzygapophyses. The postzygapophyses do not articulate with corresponding prezygapophyses (Fig 5C). Vertebrae II and III have transverse processes with round distal termini (Fig 5B and 5C). The transverse processes of vertebra II are connected to lightly stained ankylosed ribs by a short cartilaginous extension (Fig 5B). The neural arches of all presacral vertebrae and the sacrum are well ossified, however, they do not articulate with the vertebral centra (Fig 5C). The postsacral vertebra has two lateral ossifications along the neural arch. Whitish bodies of calcified endolymph (“lime sacs” as in Duellman & Trueb [37]) are apparent in the extradural space of vertebrae II, III and IV.

At stages 42 and 46 (Fig 5D–5F) all vertebral components are present, including presacrals, sacrum, hypochord, coccyx and transverse processes of vertebrae II–VIII. The transverse processes of vertebrae II, III and IV are curved posteriorly at their distal ends (Fig 5D–5F). Transverse processes of the vertebra III expand anterolaterally and the terminal ends are enlarged compared to others (Fig 5E). The postsacral vertebra I is present at stage 42 (Fig 5D) and is later resorbed by stage 46. The onset of notochord is initiated in the sacral region with a large depression. The notochord is indistinguishable by stage 46, however, the coccyx and the hypochord have still not fused to form the urostyle (Fig 5F). The calcified endolymphatic sacs persist through stages 42 and 46 (Fig 5D–5F). These irregular-shaped whitish globules extend in parallel within the vertebral column posterior to the auditory capsules. The paired ribs on vertebra II are fully ankylosed to the transverse processes by stage 46 (Fig 5E and 5G).

## Discussion

This description of the external morphology, osteology and ecological adaptations, of *Micrixalus herrei* provides the first confirmed report of a tadpole from the ancient anuran family, Micrixalidae. The present study also bridges a significant gap by documenting the life history of at least one representative species from each of the world’s 54 anuran families, which facilitates comparative studies across many disciplines, including development, evolution, and ecology.

There is a single report of *Micrixalus* tadpoles by Smith [14] based on “two poorly preserved specimens said to belong to *M*. *opisthorhodus*” (currently *M*. *phyllophilus*). According to the basic description provided by Smith [14], these tadpoles bear a single row of keratodonts, which subsequently were considered “characteristic” of micrixalid tadpoles without further investigation [15–17]. This observation is contrary to our finding that *M*. *herrei* tadpoles, along with three other micrixalid species (currently being studied in detail), lack keratodonts (tooth rows). Given that many features of the tadpoles described by Smith [14] are synapomorphies with larvae of many other groups, we presume that this description is erroneous. Furthermore, Smith [14] does not mention the fossorial habitat of the two tadpoles, which is their most conspicuous feature. Unfortunately, Smith’s [14] description could not be validated due to the loss of his specimens. We have failed to recover them from any potential museum collections (Natural History Museum, London and Zoological Survey of India, Kolkata).

The unusual morphological characters of *Micrixalus herrei* show convergence with the fossorial forms that are currently known globally. Here we discuss some of the unique features and morphological adaptations of *Micrixalus herrei* tadpoles that correlate with a fossorial habit.

### External morphology

*Micrixalus herrei* tadpoles wriggle back into the gravel beds as soon as they are released from captivity, suggesting that they are active and obligate burrowers. They possess a suite of characters shared with disparate fossorial lineages across the Neobatrachia. One consistent feature, eel-like bodies, is known to be associated with active burrowing into sand and for moving through gravel and vegetation [e.g., 21–22,38–42]. Furthermore, this form enables fossorial larvae to enter crevices of various sizes and shapes [23]. Muscularized tails with reduced tail fins are also common to *M*. *herrei*, *Leptobrachella mjobergi* [23], *Otophryne pyburni* [21], *Nymphargus grandisonae* [42], *Espadarana andina* [39], *Hyalinobatrachium ibama* [43], *Centrolene hesperium* [38], *Tetrohyla pulverata* [40], *Cardioglossa manengouba* [41,44], *Staurois tuberlinguis* and *S*. *natator* [45], and is assumed to enable locomotion within a sandy substrate [46].

Altig & Johnston [1] defined ecomorphological guilds of “aquatic, non-reproductive, energy gathering” anuran larvae based on the oral disc and body morphologies. Existence of various energy sources in different microhabitats enables resource partitioning and a subsequent variety of morphotypes of exotrophic larvae. Fossoriality, one such ecomorph guild, was configured as “type I” and “type II” based on the presence or absence of tooth rows [1]. Most centrolenids and ranids (*Staurois natator*, *S*. *tuberilinguis*, *S*. *guttatus*) bear tooth rows and fall under the category “type I.” Contrastingly, micrixalids (*Micrixalus herrei*), megophryids (*Leptobrachella mjobergi*), arthroleptids (*Cardioglossa manengouba*, *C*. *melanogaster*, *C*. *pulchra*) and microhylids (*Otophryne pyburni*, *O*. *steyermarki*) lack tooth rows and fall under “type II.” These variations in tooth rows correspond with food resources within lotic habitats [1]. Additionally, the funnel-shaped oral discs of fossorial larvae differ in the extent to which they bear papillae. *Otophryne pyburni*, *O*. *steyermarki*, *Cardioglossa manengouba*, *C*. *melanogaster*, *C*. *pulchra* have papillae only around the lower labium. Centrolenids have papillae demarcating the complete oral disc except for the medial gap on the upper labium, whereas *M*. *herrei* has complete rows of papillae around the entire oral disc.

Jaw sheaths are absent in *Leptobrachella mjobergi* [23], however, robust serrated upper and lower sheaths are observed in other fossorial tadpoles [21], including *M*. *herrei*. However, keratinous teeth of *Otophryne pyburni* are considered potentially homologous with the keratinous jaw sheaths of stream-dwelling tadpoles [21]. Even though it was suggested that the presence of these strong, serrated tooth rows/ jaw sheaths could be of a predatory significance [47], Wassersug & Pyburn [21] suggested that this feature enables the prevention of large sand grains from entering the mouth by acting as a “sieve.” *Micrixalus herrei* tadpoles are apparently less exclusive of sand and accompanying detritus in their feeding. The organic matter found in sand is apparently a source of energy in their nutrient-poor environments.

Wassersug & Pyburn [21] consider long spiracular tube to be an adaptation to generate passive water currents among fossorial tadpoles that facilitate respiration and feeding. Long spiracles are seen in most fossorial larvae (e.g., *Otophryne pyburni*, *O*. *steyermarki*, *Leptobrachella mjobergi*, *Cardioglossa manengouba*, *C*. *melanogaster*, *C*. *pulchra*, *M*. *herrei*), however, their length vary by developmental stage and between species [21,41,48,49]. We did not observe long spiracular tubes in *M*. *herrei*, suggesting an inconsistent convergence of this generalized fossorial tadpole feature.

Fossorial forms also have small, non-bulging, and distinctly parasagittal eyes [1]. However, this varies in some species as a result of ontogenetic variations. In centrolenids, rudimentary eye pigmentation occurs in early developmental stages (Gosner stages 25–27) but large functional eyes are apparent in older tadpoles [39,42,43,50–53], which is similar to our observations in *Micrixalus herrei* (Fig 4A–4F). As the eyes of *M*. *herrei* develop (after stage 30), they become overlaid with thickened epidermis (Fig 4G and 4I), which likely protects them from abrasion.

*Micrixalus herrei*, centrolenids, *Cardioglossa* spp and *Staurois* spp [39,41–45,50–55] have a red tinge to their lightly pigmented bodies (ventral sides in the case of *M*. *herrei*) due to an extensive capillary network beneath some areas of the skin. Evidence suggests that this network is habitat-specific. In the centrolenid, *Cochranella granulosa*, tadpoles with red-tinged bodies occupy hypoxic microhabitats, whereas tadpoles with pale bodies inhabit well-oxygenated water [53]. The plasticity of this network of capillaries could be facilitating increased cutaneous gas exchange in oxygen-poor habitats.

Ontogenetic size variation within a specific developmental stage is evident in centrolenids [39,42,51] and *Micrixalus herrei*. For example, in *Cochranella granulosa*, larger and more active tadpoles were observed in hypoxic environments [53]. Though we did not test for oxygen concentrations in this study, we observed that larger individuals lived in deeper sand compared to smaller ones.

Lateral expansion of the skin on ventral sides of the bodies as observed in *Cardioglossa manengouba*, *C*. *melanogaster* and *C*. *pulchra* [44] was also evident in *M*. *herrei*. Though the function of this is unknown, it is thought to be facilitating the undulating movements of the tadpoles underground [44].

### Internal morphology

Double-stained skeletal preparations of *Micrixalus herrei* reveal similarities and remarkable differences with the fossorial larvae of *Leptobrachella mjobergi* [23], *Otophryne pyburni* [21] and *Cardioglossa manengouba* [41]. According to Wassersug & Pyburn [21], burrowing and movement through interstitial spaces in sand and gravel corresponds with a dorsoventrally flattened and dorsolaterally expanded cranium, and well-developed suprarostral cartilages in *Otophryne pyburni*. These characteristics of the chondrocranium are evident in *Micrixalus herrei* and *Leptobrachella mjobergi* [23] and likely perform the same functions. The notochord usually extends from the anterior margins of the otic capsules towards the tip of the tail [56]. However, in *Micrixalus herrei* and *Leptobrachella mjobergi* it does not extend to the basal plate anteriorly, but terminates before the atlantal cotyle-occipital condyle junction. This is assumed to be an adaptation to increase head maneuverability [23]. A greater range of lateral head movements may help dislodge sand particles while the body and tail generate propulsive force.

The postcranial skeleton of *Micrixalus herrei* also has several unique features. *Leptobrachella mjobergi* has supernumerary ossified caudal vertebrae associated with its fossorial lifestyle [23,57], however, these were not observed in *Micrixalus herrei*. Whitish globular calcified endolymphatic sacs are abundant in *M*. *herrei*. Extensive endolymphatic sacs are a common observation among developing larval stages that have been recorded elsewhere and are thought have a function in storage of calcium carbonate as aragonite [58–62]. However, these have not been previously reported in other fossorial tadpoles, except for the densely calcified deposits observed in cranial endolymphatic sacs of *Cardioglossa manengouba* [41]. These sacs, which are often associated with the auditory capsules, have posterior extensions along the vertebral column and increase in size during development in *M*. *herrei*. However, we have found no similar descriptions of these extensions along the vertebral column reaching posteriorly to the sacrum, as in *M*. *herrei*. During metamorphic climax, when some tadpoles are non-feeding, calcium carbonate stored in these sacs may be utilized for ossification [37,59], but the function of postcranial endolymphatic extensions is unknown. However, they may reflect an increased mineral supply for metamorphic ossification, which could also be an additional benefit of the supernumerary vertebrae of *Leptobrachella mjobergi*.

Ribs are known to occur in only four anuran families—Leiopelmatidae, Discoglossidae, Dicroglossidae and Pipidae [37,63–66]—and are usually associated with the first few vertebrae (Presacrals II–IV); these families, however, do not have fossorial tadpoles. *Micrixalus herrei* is the only fossorial tadpole known to have ankylosed ribs. These could be functioning together with the well-developed myotomes to facilitate the eel-like locomotion or provide protection for internal organs while moving through sand.

### Evolutionary and conservational perspectives

Many aspects of the biology of *Micrixalus herrei* tadpoles, which we have presented here, together with information on three other tadpoles of *Micrixalus* species (SDB personal observation), may be representative of the entire family. Tadpoles of three other *Micrixalus* species (possibly *M*. *fuscus*, *M*. *nelliyampathi*, and *M*. *phyllophilus*) have been found at similar sites where adults are locally abundant. The biology of these three tadpoles is currently being studied.

The documentation of *Micrixalus herrei* larval habitat informs decisions relating to their conservation and management. For instance, their association with forest cover, perennial streams, and sandy banks reveals specific habitat requirements for this species and potentially other threatened *Micrixalus* (e.g., *M*. *gadgili* and *M*. *kottigeharensis*). These tadpoles are also likely obligate burrowers that breed during low-water periods. This habitat and breeding information provides a basis for comparison with other *Micrixalus* species, most of which were only recently described [8].

Fossorial tadpoles have evolved multiple times independently in disparate evolutionary lineages. We have only a limited understanding of the evolutionary and ecological reasons for fossoriality of *Micrixalus* tadpoles. Site fidelity within a single genus or family can be driven by a number of factors. Adult micrixalids consistently associate with stream-side habitats. This may, in part, be due to the habitat requirements of their embryos and larvae, similar to many other anuran species (e.g., the oophagous larvae of *Frankixalus jerdonii* that require water-filled tree holes [67]). Rapid fluctuations of surface water in streams during the pre and post-monsoon periods of the Western Ghats, extreme within-stream predator pressure, or the abundance of sub-surface food may each partially account for the fossoriality in this family. These initial observations of *Micrixalus* will help build a knowledge base that will be important for determining the unique drivers of this ecomorphological guild and the monitoring of this ancient and enigmatic family of frogs.

## Supporting Information

S1 Table Fossorial tadpoles recorded from five of the anuran families—Arthroleptidae, Centrolenidae, Megophryidae, Microhylidae and Ranidae—and their corresponding references. (DOCX)
