# Supplementary material for: Unearthing the Fossorial Tadpoles of the Indian Dancing Frog Family Micrixalidae
Source: PLoS One. 2016 Mar 30;11(3):e0151781. doi: 10.1371/journal.pone.0151781 (PMC4814069; doi:10.1371/journal.pone.0151781)
Supplement: S1 Table — (DOCX) [file pone.0151781.s001.docx]

**Table S1: Some of the fossorial tadpoles recorded from five of the anuran families— Arthroleptidae, Centrolenidae, Megophryidae, Microhylidae and Ranidae—and their corresponding references.**

| Family | Species | References |
| --- | --- | --- |
| Centrolenidae | *Centrolene hesperium* | Cadle & McDiarmid, 1990 |
|  | *Centrolene savagei* | Diaz-Gutierrez *et al.* 2013 |
|  | *Centrolene daidaleum* | Rada *et al.* 2007 |
|  | *Cochranella* *granulosa* | Hoffmann, 2010 |
|  |  | Starrett, 1960 |
|  |  | Hoff *et al,* 1999 |
|  | *Cochranella* *resplendens* | Teran-Valdez *et al,* 2009 |
|  | *Espadarana andina* | Mijares-Urrutia, 1990 |
|  | *Espadarana* *prosoblepon* | Starrett 1960 |
|  | *Hyalinobatrachium aureoguttatum* | Teran-Valdez *et al,* 2009 |
|  | *Hyalinobatrachium cappellei* | Noonan & Bonett, 2003 |
|  | *Hyalinobatrachium colymbiphyllum* | Jaramillo *et al,* 1997 |
|  | *Hyalinobatrachium fleischmanni* | Starrett, 1960 |
|  |  | Wassersug & Hoff, 1979 |
|  |  | Villa & Valerio, 1982 |
|  | *Hyalinobatrachium ibama* | Rada *et al* 2007 |
|  | *Nymphargus grandisonae* | Ospina-Sarria *et al* 2011 |
|  | *Teratohyla pulverata* | Hoffmann, 2004 |
|  | *Teratohyla spinosa* | Starrett, 1960 |
|  | *Vitreorana eurygnatha* | Heyer, 1985 |
|  | *Vitreorana ritae* | Menin *et al,* 2009 |
| Microhylidae | *Otophryne pyburni* | Pyburn, 1980 |
|  |  | Wassersug & Pyburn, 1987 |
|  |  | Campbell & Clarke, 1998 |
|  |  | MacCulloch *et al,* 2008 |
|  |  | Hoff *et al,* 1999 |
|  | *Otophryne steyermarki* | MacCulloch et al, 2008 |
|  | *Scaphiophryne gottlebei* | Mercurio & Andreone, 2006 |
| Megophryidae | *Leptobrachella mjobergi* | Haas *et al,* 2006 |
|  |  | Inger, 1985 |
|  |  | Handrigan *et al,* 2007 |
|  | *Leptolax* spp. | Inger, 1985 |
|  |  | Handrigan *et al,* 2007 |
|  |  | Wells, 2007 |
| Arthroleptidae | *Cardioglossa leucomystax* | Lamotte, 1961 |
|  | *Cardioglossa manengouba* | Blackburn, 2008 |
|  |  | Hirschfeld *et al,* 2012 |
|  | *Cardioglossa melanogaster* | Hirschfeld *et al,* 2012 |
| Ranidae | *Staurois guttatus* | Haas & Das, 2012 |
|  |  | Preininger *et al*, 2012 |
|  | *Staurois natator* | Malkamus *et al,* 1999 |
|  | *Staurois tuberlinguis* | Malkamus *et al,* 1999 |
|  | *Staurois parvus* | Preininger *et al,* 2012 |
|  | *Staurois spp* | Inger & Wassersug, 1990 |

References:

1. Cadle JE, McDiarmid RW. Two new species of Centrolenella (Anura: Centrolenidae) from northwestern Peru. Proc Biol Soc Wash. 1990; 103:746–768.

2. Diaz-Gutierrez N, Vargas-Salinas F, Rivera-Correa M, Rojas-Morales JA, Escobar-Lasso S, Velasco JA, Gutierrez-Cardenas PDS, Amezquita A. Description of the previously unknown advertisement call and tadpole of the Colombian endemic glassfrog *Centrolene savagei* (Anura: Centrolenidae). Zootaxa. 2012; 3686: 289–296

3. Rada M, Ruedad-Almonacid JV, Velasquez-Álvarez AA, Sanchez-Pacheco SJ. 2007. Descripción de las larvas de dos Centrolénidos (Anura: Centrolenidae) del noroccidente de la cordillera oriental, Colombia. Pap Avulsos Zool. 2007; 47: 259−272.

4. Hoffmann H. Cyanosis by Methemoglobinemia in Tadpoles of *Cochranella granulosa* (Anura: Centrolenidae). Rev. Biol. Trop. (Int. J. Trop. Biol.). 2010; 58: 1467−1478.

5. Starrett P. Descriptions of tadpoles of middle American Frogs. Museum of Zoology: University of Michigan; 1960. pp. 11−17.

6. Hoff K, Blaustein AR, McDiarmid RW, Altig R. Behavior, Interactions and Their Consequences. In: McDiarmid RW, Altig R, editors. Tadpoles, the biology of anuran larvae. Chicago: University of Chicago Press; 1999. pp. 215–239.

7. Teran-Valdez A, Guayasamin JM, Coloma LA. Description of the tadpole of *Cochranella resplendens* and redescription of the tadpole of *Hyalinobatrachium aureoguttatum* (Anura, Centrolenidae). Phyllomedusa. 2009; 8: 105−124.

8. Mijares-Urrutia A. The tadpole of *Centrolenella andina* (Anura: Centrolenidae). J Herpetol. 1990; 24: 410–412.

9. Noonan B, Bonett R. A new species of *Hyalinobatrachium* (Anura: Centrolenidae) from the highlands of Guyana. J Herpetol. 2003; 37: 91–97.

10. Jaramillo FE, Jaramillo CA, Ibanez DR. Renacuajo de la rana de cristal *Hyalinobatrachium colymbiphyllum* (Anura: Centrolenidae). Rev Biol Trop. 1997; 45: 867–870.

11. Wassersug RJ, Hoff K. A Comparative study of the buccal pumping mechanism of tadpoles. Biol J Linn Soc. 1979; 12: 225–259.

12. Villa J, Valerio CE. Red, white and brown preliminary observations on the color of the centrolenid tadpole (Amphibia: Anura: Centrolenidae). Brenesia. 1982; 19: 1–16.

13. Ospina-Sarria JJ, Bolívar-GW, Mendez-Narvaez J, Burbano-Yandi C. The tadpole of *Nymphargus grandisonae* (Anura, Centrolenidae) from Valle del Cauca, Colombia. South Am J Herpetol. 2011; 6: 79−86.

14. Hoffmann H. Description if the previously unknown tadpole of *Hyalinobatrachium pulveratum* (Anura: Centrolenidae). Rev Biol Trop. 2004; 52: 219–228.

15. Heyer WR. Taxonomic and natural history notes of frogs of the genus *Centrolenella* (Amphibia: Centrolenidae) from southeastern Brasil and adjacent Argentina. Pap Avulsos Zool. 1985; 36: 1−21.

16. Menin M, A. P. Lima AP, D. J. Rodriguez DJ. The tadpole of *Vitreorana oyampiensis* (Anura, Centrolenidae) in Central Amazonia, Brazil. Zootaxa. 2009; 2203: 65−68.

17. Pyburn WF. An unusual anuran larva from the Vaup.s region of southeastern Colombia. Pap Avulsos Zool. 1980; 33: 231−238.

18. Wassersug RJ, Pyburn WF. The biology of the pe-ret’ toad, *Otophryne robusta* (Microhylidae), with special consideration of its fossorial larva and schematic relationships. Zool J Linn Soc. 1987; 91: I37–69.

19. Campbell JA, Clarke BT. A review of frogs of the genus *Otophryne* (Microhylidae) with the description of a new species. Herpetologica. 1998; 54: 301–317.

20. MacCulloch RD, Lathrop A, Minter LR, Khan SZ. *Otophryne* (Anura: Microhylidae) from the highlands of Guyana: redescriptions, vocalizations, tadpoles and new distributions. Pap Avulsos Zool. 2008; 48: 247–261.

21. Mercurio V, Andreone F. The tadpoles of *Scaphiophryne gottlebei* (Microhylidae, Scaphiophryninae) and *Mantella expectata* (Mantellidae, Mantellinae) from Isalo Massif, central-southern Madagascar. Alytes. 2006; 23: 81–95.

22. Haas A, Hertwig S, Das I. Extreme tadpoles: The morphology of the fossorial megophryid larva, *Leptobrachella mjobergi*. Zoology. 2006; 109: 26–42.

23. Inger RF. Tadpoles of the forested regions of Borneo. Fieldiana. 1985; 26:

1–89.

24. Handrigan GR, Haas A, Wassersug RJ. Bony-tailed tadpoles: the development of supernumerary caudal vertebrae in larval megophryids (Anura). Evol Dev. 2007; 9: 190–202.25. Wells KD. 2007. The Ecology and Behavior of Amphibians. Chicago: University of Chicago Press.

26. Lamotte M. Contribution à l’étude des Batraciens de l’Ouest africain. XII.-Les formes larvaires de *Cardioglossa leucomystax* Blgr. Bull Inst Fond Afr Noire Ser A, 1961; 23: 211–216.

27. Blackburn DC. A new species of *Cardioglossa* (Amphibia: Anura: Arthroleptidae) endemic to Mount Manengouba in the Republic of Cameroon, with an analysis of morphological diversity in the genus. Zool J Linn Soc. 2008; 154: 611–630.

28. Hirschfeld M, Barej MF, Gonwouo NL, Ro’del M-O. Tadpole descriptions of three *Cardioglossa* species from southwestern Cameroon (Amphibia: Anura: Arthroleptidae). Salamandra. 2012; 48: 147−156.

29. Haas A, Das I. Frogs of Borneo — The Frogs of East Malaysia and their Larval Forms: An Online Photographic Guide. Zoological Museum Hamburg,

Germany. [Online]. 2012; Available: http://www.frogsofborneo.org.

30. Preininger D, Weissenbacher A, Wampula T, Hödl W. The conservation breeding of two foot-flagging frog species from Borneo, *Staurois parvus* and *Staurois guttatus*. Amphib Reptile Conserv. 2012; 5: 45–56(e51).

31. Malkmus R, Kosuch J, Kreutz J. Die larve von *Staurois tuberilinguis* Boulenger, 1918. Eine neuecentroleniden Kaulquappe aus Borneo (Anura: Ranidae). Herpetozoa. 1999; 12: 17−22.

32. Inger RF, Wassersug RJ. A Centrolenid-Like Anuran Larva from Southeast Asia. Zool Sci. 1990; 7: 557−561.
